# Systematic Characterization of the Disruption of Intestine during Liver Tumor Progression in the *xmrk* Oncogene Transgenic Zebrafish Model

**DOI:** 10.3390/cells11111810

**Published:** 2022-05-31

**Authors:** Yan Li, Ai Qi Lee, Zhiyuan Lu, Yuxi Sun, Jeng-Wei Lu, Ziheng Ren, Na Zhang, Dong Liu, Zhiyuan Gong

**Affiliations:** 1 Department of Biological Sciences, National University of Singapore, Singapore 117543, Singapore; aiqi@nus.edu.sg (A.Q.L.); dbs_luzhiyuan@visitor.nus.edu.sg (Z.L.); e0437708@u.nus.edu (Y.S.); jengweilu@gmail.com (J.-W.L.); e0004248@u.nus.edu (Z.R.); e0321277@u.nus.edu (N.Z.); 2 Animal Nutrition Institute, Sichuan Agricultural University, Chengdu 611130, China; 3 Department of Biology, Southern University of Science and Technology, Shenzhen 518055, China; liud@sustech.edu.cn

**Keywords:** HCC, liver tumor, intestine, gut–liver axis, cancer cachexia, zebrafish

## Abstract

The crosstalk between tumors and their local microenvironment has been well studied, whereas the effect of tumors on distant tissues remains understudied. Studying how tumors affect other tissues is important for understanding the systemic effect of tumors and for improving the overall health of cancer patients. In this study, we focused on the changes in the intestine during liver tumor progression, using a previously established liver tumor model through inducible expression of the oncogene *xmrk* in zebrafish. Progressive disruption of intestinal structure was found in the tumor fish, displaying villus damage, thinning of bowel wall, increase in goblet cell number, decrease in goblet cell size and infiltration of eosinophils, most of which were observed phenotypes of an inflammatory intestine. Intestinal epithelial cell renewal was also disrupted, with decreased cell proliferation and increased cell death. Analysis of intestinal gene expression through RNA-seq suggested deregulation of genes related to intestinal function, epithelial barrier and homeostasis and activation of pathways in inflammation, epithelial mesenchymal transition, extracellular matrix organization, as well as hemostasis. Gene set enrichment analysis showed common gene signatures between the intestine of liver tumor fish and human inflammatory bowel disease, the association of which with cancer has been recently noticed. Overall, this study represented the first systematic characterization of the disruption of intestine under the liver tumor condition and suggested targeting intestinal inflammation as a potential approach for managing cancer cachexia.

## 1. Introduction

Liver cancer, the sixth most common cancer and the fourth leading cause of cancer-related deaths worldwide, remains a global health challenge [1]. Hepatocellular carcinoma (HCC), which begins in hepatocytes, is the most common type of liver cancer. Extensive studies have shown that tumor occurrence and progression is influenced by the tumor microenvironment, which consists of secreted factors, extracellular matrix, blood vessels, as well as resident and infiltrating host cells, such as stromal cells and immune cells [2]. In addition to the interaction with the local microenvironment, tumors can cause systemic effects in other organs. For instance, tumor-derived cytokines and microvesicles could be released into circulation and reach distant organs to form pre-metastatic niches [3,4]. Moreover, tumors may cause systemic inflammation and DNA damage in distant organs, such as the gastrointestinal tract and skin [5,6]. Cancer cachexia, a syndrome characterized by weakness in multiple organs and substantial loss of skeletal muscle and adipose tissue, occurs in up to 85% of cancer patients [7]. These systemic effects of tumors, which impair host organs and significantly contribute to cancer-related deaths, remain understudied. An investigation of tumor systemic effects may provide important information on the development of therapeutic strategies for improving the health of cancer patients and survival rates.

The liver and intestine are two anatomically and physiologically connected organs that communicate extensively, a relationship called the gut–liver axis [8]. The liver releases bile acids and other bioactive mediators including antibodies and metabolites that reach the intestine through the biliary tract and circulation. These liver factors regulate nutrient absorption and metabolism, as well as intestinal microbiota and permeability. On the other hand, intestinal factors, such as dietary and microbial metabolites, regulate bile acid synthesis and glucose and lipid metabolism in the liver through the portal vein and systemic circulation. Homeostasis of the gut–liver axis is often disrupted under liver disease conditions. Many studies have shown that under alcoholic liver disease, non-alcoholic fatty liver disease and cirrhosis conditions, intestinal microbiota is altered, and intestinal barrier is disrupted, which further worsen liver inflammation and fibrosis [9]. Under the HCC condition, the change in intestinal microbiota and the importance of intestinal microbiota on HCC progression through modulating inflammation have also been documented [10,11]. Despite these studies on gut dysbiosis, there has been no systematic investigation on the histological, molecular or functional disruption of the intestine during liver disease, especially during liver tumorigenesis.

The zebrafish provides an important animal model system for the study of human cancers, especially liver cancer [12,13,14]. Our group had established several inducible HCC models in zebrafish by transgenic expression of oncogenes and had shown that they are molecularly similar to human HCC [15,16,17,18,19,20]. HCC usually developed with 100% incidence within a few weeks of oncogene induction in these fish [21,22], providing an excellent platform to investigate the systemic effects of HCC. Previously, we investigated muscle wasting, a major cancer cachexia symptom, during liver tumor progression and reported the importance of leptin signaling in muscle wasting [23]. Recently, we showed that inducing intestinal inflammation using dextran sulfate sodium (DSS) could also promote HCC progression and metastasis [24].

Zebrafish are stomachless, and their intestine connects directly to the esophagus. The intestinal architecture is largely conserved between zebrafish and mammals. The zebrafish intestine also has a mucosa layer of epithelium and lamina propria, the muscularis layer and the outermost serosa layer, while lacking the muscularis mucosa and submucosal layer [25]. Similar to that of mammals, the intestinal epithelium of zebrafish is also composed of enterocytes for absorption, goblet cells for mucus production and enteroendocrine cells that secrete hormones and peptides, with the exception of Paneth cells, which have not been identified in zebrafish. The intestinal epithelium form irregular folds which are comparable to the finger-like villi in mammals. The conservation of gene expression and regulation along the length of intestine between zebrafish and mammals has also been demonstrated [26,27]. The zebrafish model has been frequently used to study human intestinal disorders [28]. In this study, we systematically characterized the effect of liver cancer on the intestine at different stages of tumorigenesis in the *xmrk* (activated epidermal growth factor receptor homolog) transgenic zebrafish [15], through histological, cytological and transcriptomic methods.

## 2. Material and Methods

### 2.1. Zebrafish Maintenance and Doxycycline Treatment

The *xmrk* transgenic line, *Tg(fabp10:rtTA; TRE:xmrk; krt4:GFP),* was previously established in our laboratory [15]. Adult male wild-type (wt) and *xmrk* zebrafish were used in this study. Fish were immersed in 60 μg/mL doxcycline (D9891, Sigma-Aldrich, St. Louis, MO, USA) for 4–6 weeks to induce continuous expression of the *xmrk* oncogene and HCC formation [15,22]. Ten fish were collected from each group (wt and *xmrk*) at each time point (4 weeks and 6 weeks). All study protocols involving zebrafish were approved by the Institutional Animal Care and Use Committee of National University of Singapore.

### 2.2. Sample Collection and Histological, Immunohistochemistry and TUNEL Assays

Zebrafish gut samples were fixed in 10% neutral-buffered formalin solution (HT501128, Sigma-Aldrich, St. Louis, MO, USA) for 24 h, followed by dehydration, clearing and paraffin embedding. The paraffin-embedded tissues were sectioned at 5 μm in thickness using a microtome. Haematoxylin and eosin (H&E) staining was performed using standard protocols and Alcian blue-periodic acid-Schiff (AB-PAS) staining was conducted following the manufacturer’s instructions (ab245876, Abcam, Cambridge, UK). Primary and secondary antibodies used for immunofluorescent staining were mouse anti-PCNA (sc-56, Santa Cruz Biotechnology, Dallas, TX, USA) and Alexa Fluor^®^ 488 anti-mouse (A11029, Invitrogen, Waltham, MA, USA), respectively. TUNEL assay was conducted using the in situ cell death detection kit (11684795910, Roche, Basel, Switzerland) and augmented by immunostaining using anti-fluorescein CF^TM^ 488A (SAB4600050, Sigma-Aldrich, St. Louis, MO, USA).

### 2.3. Imaging and Analysis of Histological, Immunohistochemistry and TUNEL Samples

H&E and AB-PAS samples were imaged using a Zeiss AXIO Imager M2 compound microscope. An LSM900 Confocal Zeiss microscope was used to image immunofluorescent-stained samples, which were then analyzed using Fiji software [29]. The data were put through a Shapiro–Wilk normality test and a Levene test for equality of variances. Based on the normality and variance homogeneity of the datasets, Student’s t-test, Welch’s t-test, or Mann–Whitney U-test were applied to determine statistical significance. Student’s t-test was used for ordinal datasets.

### 2.4. RNA Extraction, Library Preparation and Sequencing

The whole intestines from three fish were pooled and subjected to total RNA extraction using TRIzol reagent (15596018, Invitrogen, Waltham, WA, USA). There were three biological replicates for both wt and *xmrk* fish. Total RNA samples were submitted to BGI Group, Hong Kong, for quality control verification, stranded mRNA library preparation and sequencing using DNBSEQ platform. For library preparation, mRNA was purified using Oligo (dT) beads and fragmented, followed by first-strand cDNA synthesis using random hexamer priming and second-strand cDNA synthesis using dUTP instead of dTTP. After a series of terminal repair, 3′ adenylation, adaptor ligation and degradation of the dUTP-marked strand by uracil-DNA glycosylase, the remaining strand was PCR amplified to generate the cDNA library. The cDNA library was then subjected to single-strand separation, cyclization, DNA nanoball synthesis and sequencing on DNBSEQ PE150 platform.

### 2.5. Bioinformatics Analysis of Sequencing Data

The raw reads were filtered by removing reads of low quality, adaptor contamination and high level of N bases using SOAPnuke software [30]. The filtered clean reads were aligned to zebrafish reference genes using Bowtie2 [31]. Transcript per million (TPM) gene expression level was calculated using RSEM [32]. Average TPM cutoff of 1 was applied to capture meaningfully expressed transcripts. The zebrafish gene symbols were mapped to human orthologs using ZFIN orthologous database. Differential expression between *xmrk* and wt intestine was detected using DEseq2 [33]. Genes with fold change >2 and adjusted *p*-value < 0.05 were identified as differentially expressed genes (DEGs).

Principal component analysis (PCA) was performed using the prcomp function in R and plotted using the ggplot package. The ComplexHeatmap package in R was used for hierarchical clustering by Euclidean distance and visualization [34]. Gene set enrichment analysis (GSEA) was performed using the Broad Institute GSEA software (v4.2.3, Broad Institute, MA, USA). against the Reactome pathway gene sets and hallmark gene sets from the Molecular Signature Database (MSigDB) [35].

## 3. Results

### 3.1. Morphological and Cellular Disruption of Intestine in HCC Fish

**Figure 1:**
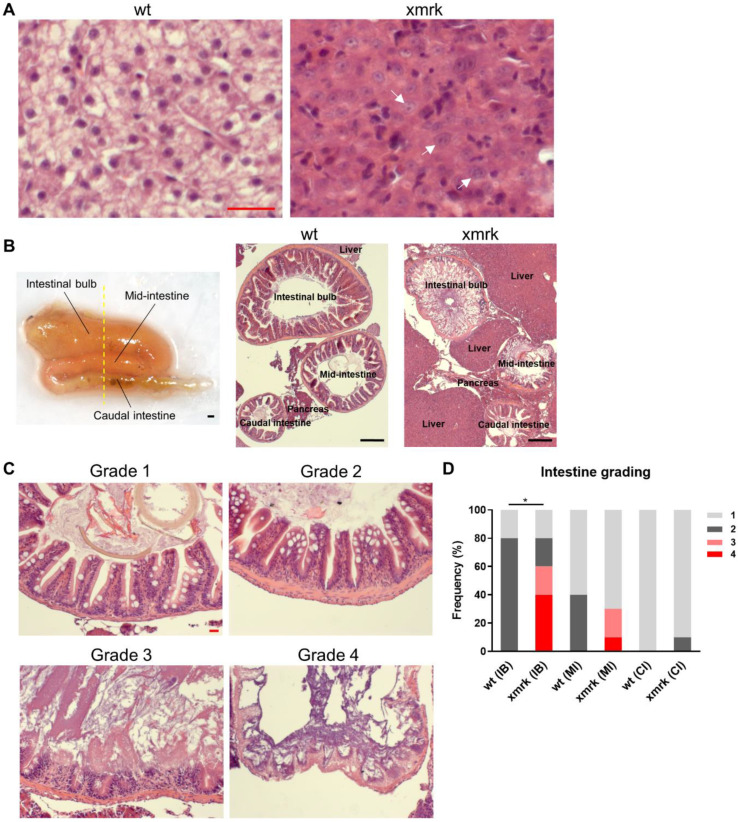
Disruption of intestine morphology after 6 weeks of HCC induction. (**A**) Representative H&E images showing the normal liver in wild-type (wt) fish and the HCC liver in *xmrk* fish. It was found that 100% of *xmrk* livers progressed into HCC after 6 weeks of doxycycline treatment. White arrows indicate example tumor cells with large irregular nuclei and prominent nucleoli. (**B**) Dissected intestine showing the folding of intestine into three segments (left panel). Intestine samples were sectioned transversely in order to view all three segments of the intestine concurrently on the same section. The yellow dashed line showed the approximate position of section. Representative H&E images taken at 50× magnification showing all three intestine segments and the surrounding liver and pancreas on the same section for wt and *xmrk* fish, respectively (middle and right panels). (**C**) Representative H&E images of the intestine taken at 200× magnification. All three segments in each wt and *xmrk* intestine sample were assigned a grade based on phenotype severity, with grade 1 being the least severe and grade 4 being the most severe. (**D**) Quantification of intestine grading percentage in wt vs. *xmrk*. Grade numbers are indicated in the legend according to examples in C. Scale bar in red 20 μm, black 200 μm. IB: Intestinal bulb; MI: Mid-intestine; CI: Caudal intestine. * *p <* 0.05.

In order to investigate the effect of HCC on the intestine, *xmrk* transgenic zebrafish were treated with 60 μg/mL doxycycline. After 6 weeks of treatment, HCC was induced in 100% of *xmrk* fish (Figure 1A), characterized by the loss of hepatocyte plate structure, large irregular nuclei and prominent nucleoli, as described previously [15,22]. The adult zebrafish intestine is a tube that folds twice along the anterior–posterior body axis in the abdominal cavity and thus has three distinct segments: the proximal intestine or intestinal bulb (IB), mid-intestine (MI) and caudal intestine (CI) (Figure 1B, left panel) [25,27]. Gut samples were then sectioned transversely at the plane roughly indicated by the dashed line in Figure 1B in order to concurrently observe all three intestinal segments (Figure 1B, middle and right panels). Preliminary examination showed inter-segmental differences. Therefore, all analyses were performed separately for each segment.

Histological observation of H&E staining revealed significant changes in intestinal morphology in *xmrk* fish. Each intestinal segment in an intestine sample was assigned a grade based on the severity of morphological disruption (Figure 1C), with 1 being the least severe and 4 the most severe. Grade 1 referred to perfectly normal intestine structure with distinct and intact villi. Grade 2 intestines had distinct villi but showed mild epithelial disruption at the villus tips. Grade 3 intestine presented with “lacerated” villi, or villi sloughing, showing severe disruption. Grade 4 was assigned to samples with intense sloughing and little or no discernible structure in the intestine. The frequency of each grade in each segment and group was quantified and is presented in Figure 1D. Wild-type intestine only showed grades 1 and 2, while grades 3 and 4 were only found in *xmrk* fish. In general, there was increasing morpological damage from the anterior to posterior intestine, i.e., the intestinal bulb showed the most drastic morphological damage in *xmrk* fish compared to wt fish, whereas there was not much change in the caudal intestine.

**Figure 2:**
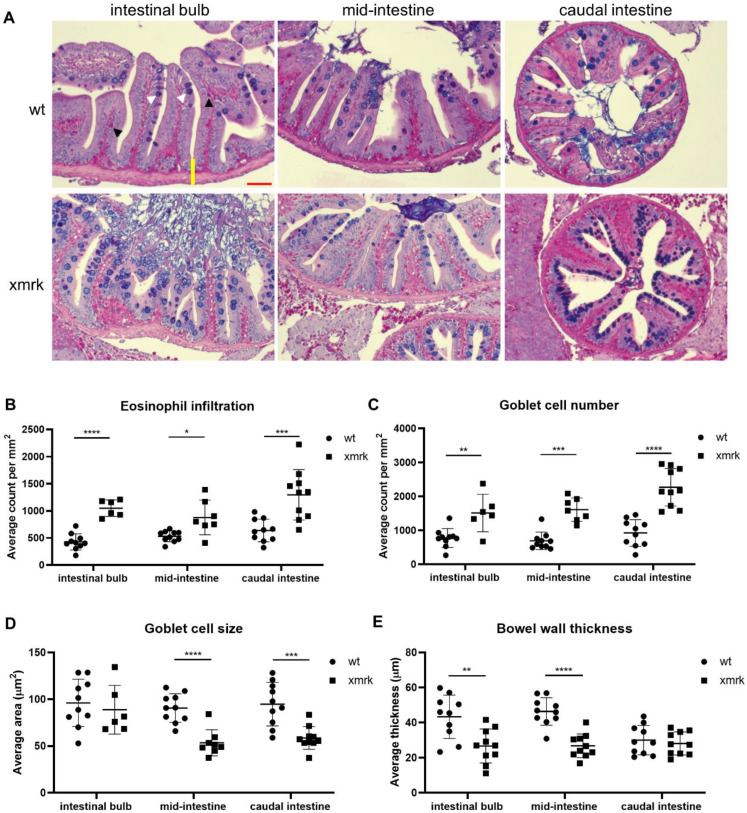
Characterization of intestine phenotype after 6 weeks of HCC induction through Alcian blue-periodic acid-Schiff (AB-PAS) stain. (**A**) Representative images of AB-PAS staining in all three intestine segments. Scale bar in red 50 μm. (**B**–**E**) Quantification of eosinophil counts (**B**), goblet cell counts (**C**), goblet cell size (**D**) and bowel wall thickness (**E**) in wt vs. *xmrk* intestine. Bowel wall was measured from the trough between two villi to the outer edge of the intestine (shown as a yellow line in the top left representative image). Black arrowheads indicate fuchsia-colored eosinophils, and white arrowheads indicate bluish-purple goblet cells. * *p <* 0.05, ** *p <* 0.01, *** *p <* 0.001, **** *p <* 0.0001.

Samples were stained using the Alcian blue-periodic acid-Schiff (AB-PAS) method to further characterize the intestinal phenotype under the HCC condition (Figure 2A). Alcian blue stains mucin-producing goblet cells, while periodic acid-Schiff stains eosinophils bright fuchsia. This method has also been previously used to observe these cell types in the zebrafish intestine [36,37,38]. Both goblet cells and eosinophils are essential for the maintenance of intestinal epithelial barrier function and intestine homeostasis [39,40], and deregulation in the number or function is associated with diseases such as inflammatory bowel disease (IBD), intestinal cancers and bacterial and parasitic infections of the intestine [39,41]. Compared to wt, *xmrk* fish showed increased eosinophil and goblet cell counts in all intestinal segments (Figure 2B,C). Goblet cells also shrunk in size in the MI and CI (Figure 2D). Additionally, bowel wall thickness was decreased in the IB and MI but unaffected in the CI (Figure 2E).

### 3.2. Disruption of Intestinal Epithelial Cell Renewal in HCC Fish

**Figure 3:**
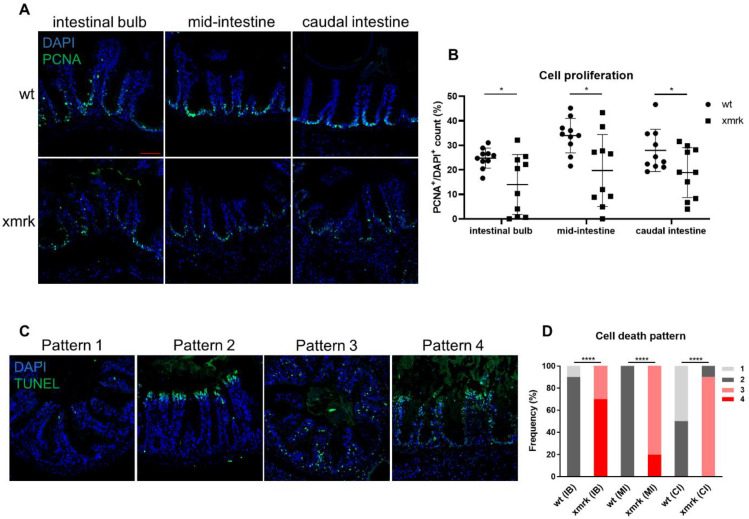
Cell proliferation and cell death in the intestine after 6 weeks of HCC induction. (**A**) Representative images of immunofluorescent staining for PCNA in the three intestine segments. (**B**) Quantification of cell proliferation in the intestine. Percentage of PCNA^+^ cell counts compared to DAPI^+^ cell counts is presented. (**C**) Representative images of TUNEL staining, classified into four different patterns. (**D**) Quantification of cell death pattern in the intestine based on frequency. Scale bar in red 50 μm. IB: intestinal bulb; MI: mid-intestine; CI: caudal intestine. * *p <* 0.05, **** *p <* 0.0001.

The intestinal epithelial cells undergo continuous renewal. Epithelial cells differentiate from stem cells at the base of villi, move to the tips, die and are shed into the lumen [25]. Cell proliferation and cell death in the intestine were examined to determine the effects of HCC on intestinal cell population dynamics. Immunofluorescent staining was performed using proliferating cell nuclear antigen (PCNA) as a cell proliferation marker, and the percentage of PCNA^+^ cell number over DAPI^+^ cell number was quantified (Figure 3A,B). The proliferating cells, which were PCNA^+^, were observed mostly at the base of villi in both wt and *xmrk* intestine. There was a significant decrease in cell proliferation in all three segments of the *xmrk* intestine compared to the wt intestine, though cell proliferation in the *xmrk* intestine was variable across samples. TUNEL assay revealed different cell death patterns in the intestine, and these were classified into four patterns, as shown in Figure 3C. Pattern 1 intestine samples had little to no cell death. The dying cells in intestines shown in pattern 2 were located at the tips of the intestinal villi, which was the most common pattern in the wt intestine. Studies in the zebrafish intestine also identified cell death occuring at villi tips [25,27]. Pattern 3 showed many dying cells dispersed throughout the epithelium and lamina propria rather than being concentrated at the villi tips. Intestines showing extensive cell death throughout the intestinal tissue were considered to have pattern 4. Patterns 1 and 2 were found almost exclusively in the wt, while Patterns 3 and 4 were present only in the *xmrk* intestine (Figure 3D). Cell death in the *xmrk* intestine seemed to be the most extensive and severe in the IB, followed by the MI and the CI.

### 3.3. Progressive Disruption of Intestine during Liver Tumor Progression

**Figure 4:**
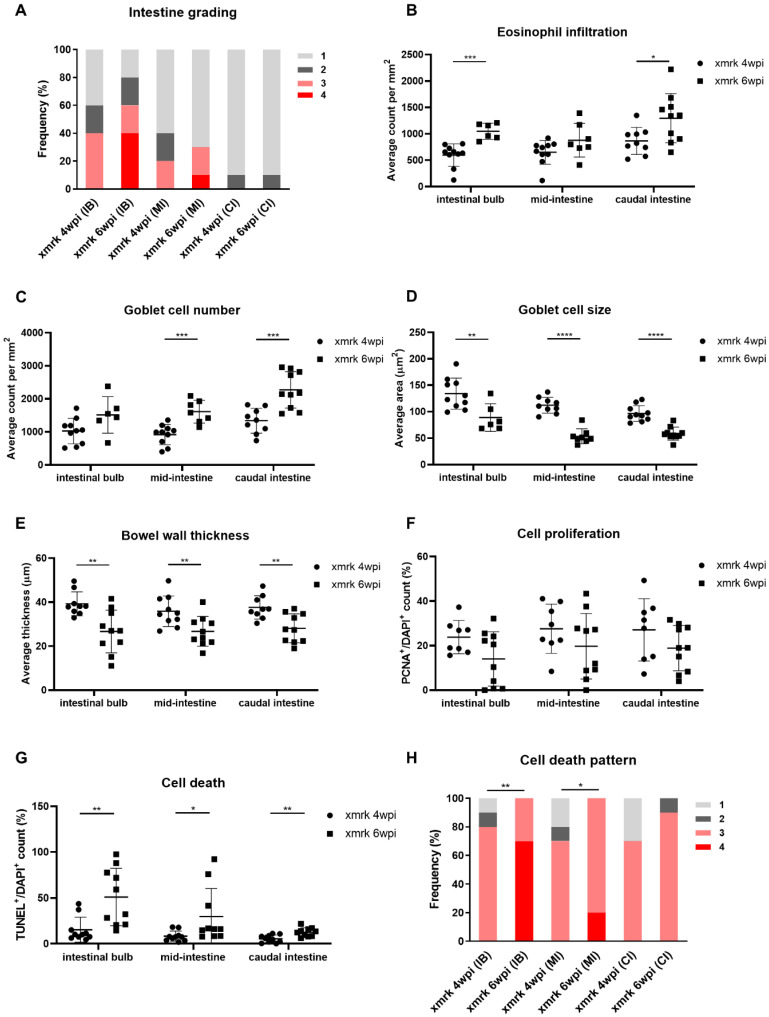
Progressive disruption of intestine structure upon HCC induction. (**A**–**H**) Quantification of intestinal severity grading (**A**), eosinophil counts (**B**), goblet cell counts (**C**), goblet cell size (**D**), bowel wall thickness (**E**), cell proliferation (**F**), cell death (**G**) and cell death pattern (**H**) in 4-week HCC (*xmrk* 4 wpi) vs. 6-week HCC (*xmrk* 6 wpi). wpi: weeks post-oncogene induction; IB: intestinal bulb; MI: mid-intestine; CI: caudal intestine. * *p <* 0.05, ** *p <* 0.01, *** *p <* 0.001, **** *p <* 0.0001.

To ascertain if the phenotype observed in the *xmrk* fish intestine is progressive with liver tumor progression, we compared the intestinal phenotype at 4 and 6 weeks post-induction (wpi). HCC penetrance was 100% in *xmrk* fish at both 4 and 6 wpi. The 4 wpi intestine underwent the same analyses as mentioned above, and all quantified data are shown in Supplementary Figure S1. The 4 wpi data generally showed similar trends to the 6 wpi data. The *xmrk* 4 wpi and *xmrk* 6 wpi data were then compared and tested for statistical significance. None of the *xmrk* intestine at 4 wpi had morphology that was considered grade 4, and more fish showed grades 1 and 2 intestine than in the *xmrk* 6 wpi (Figure 4A). Eosinophil and goblet cell counts increased from 4 wpi to 6 wpi (Figure 4B,C), and goblet cell size decreased (Figure 4D). The bowel wall was also thinner in *xmrk* 6 wpi than that in 4 wpi (Figure 4E). Cell proliferation showed an obvious decrease in the IB, while cell death increased throughout the intestine (Figure 4F,G). Cell death patterns were also less severe in the *xmrk* 4 wpi intestine, with none showing extensive pattern 4 cell death (Figure 4H). Though some datasets did not show statistical significance, the trend was noticeable and followed the trend of datasets, which were significant. Overall, these data strongly suggested that the severity of the observed *xmrk* intestine phenoype was related to the liver tumor and worsened as the tumor progressed.

### 3.4. Transcriptomic Change of Intestine in HCC Fish

**Figure 5:**
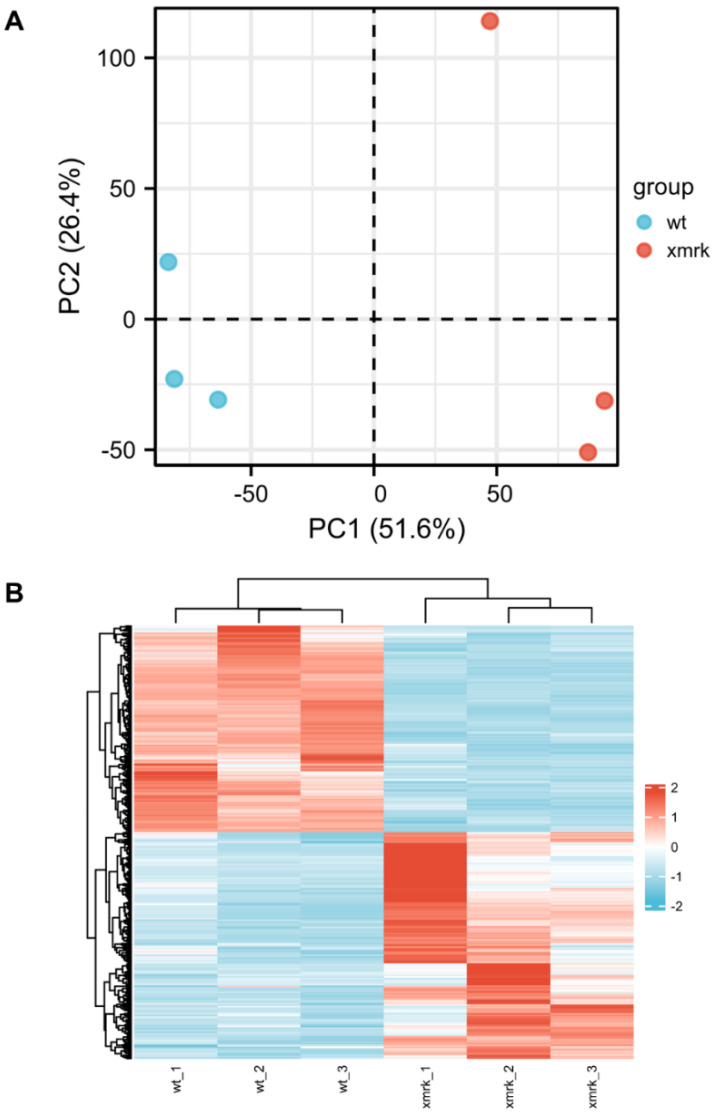
Overview of RNA─seq data. (**A**) Principal component analysis (PCA) plot of wt and *xmrk* intestine datasets. (**B**) Hierarchical clustering of wt and *xmrk* intestine datasets and heatmap using differentially expressed genes (DEGs) with average TPM > 10. Values were row scaled using z─scores to show relative expression. Blue and red indicated low and high expression, respectively.

To investigate the transcriptomic status underlying the intestine disruption in *xmrk* fish during liver tumorigenesis, whole intestines from wt and *xmrk* fish at 6 wpi were subjected to high-throughput RNA sequencing. An average of 102.83 million raw reads, which generated 87.99–89.38 million clean reads, were obtained from each of the six samples after filtering. In total, 54.24–65.3% of the clean reads could be uniquely mapped to the zebrafish reference sequence database (Supplementary Table S1), and a total of 24,754 genes were detected. In total, 14,068 genes were considered meaningfully expressed, as they had an average transcript per million (TPM) >1 (Supplementary Table S2). Principal component analysis (PCA) of the global gene expression (Figure 5A) showed clear separation between the wt and *xmrk* intestine on the first principal component (PC1), which explained 51.6% of the differences. However, there was considerable variation within the *xmrk* group, as the three replicates spread along the PC2 axis. The hierarchical clustering result further supported that the *xmrk* intestine samples clustered together and were distinct from the wt intestine samples (Figure 5B). A total of 1635 differentially expressed genes (DEGs) were identified with fold change >2 and adjusted *p*-value < 0.05 between the *xmrk* and wt intestine (Supplementary Table S3). Among these DEGs, 992 were upregulated and 643 were downregulated.

To understand the transcriptomic change in *xmrk* intestine from a broad range of biological processes, gene set enrichment analysis (GSEA) of the Reactome pathways was performed. Significantly deregulated pathways are presented in Table 1, with a stringent cut-off of the absolute value of normalized enrichment score (NES) >1.5 and false discovery rate (FDR) *q*-value < 0.05. Positive NES values indicated upregulation (marked in red), and negative NES values indicated downregulation (marked in blue). The deregulated Reactome pathways were grouped into major categories based on the hierarchy from the Reactome Knowledgebase. Downregulation of pathways in cell cycle, DNA replication and DNA repair were found in the *xmrk* intestine, suggesting a decrease in cell proliferation, and this was consistent with the result of PCNA immunofluorescent staining (Figure 3A,B). Pathways of transcription, post-transcriptional processing of mRNA, translation and post-translational processing of protein were downregulated, corresponding to the arrest of cell cycle and cell growth. Many pathways of the extracellular matrix (ECM) organization were upregulated, including ECM proteoglycans, elastic fiber formation, collagen formation, syndecan interactions, laminin interactions and integrin cell surface interactions. Activation of these pathways indicated the excessive deposition of ECM components in the intestine. Meanwhile, there was an activation of matrix metalloproteinases, which is involved in the turnover of ECM components. Hemostasis pathways were also activated. Among immune systems pathways, complement cascades were activated. The complement cascades are mainly expressed in enterocytes, and hyperactivation of complement may underline chronic intestinal inflammation [42]. For metabolism pathways, cholesterol biosynthesis and biological oxidations were activated, whereas energy production was decreased.

### 3.5. Comparison of xmrk Intestine with Human Intestinal Disease Conditions

**Figure 6:**
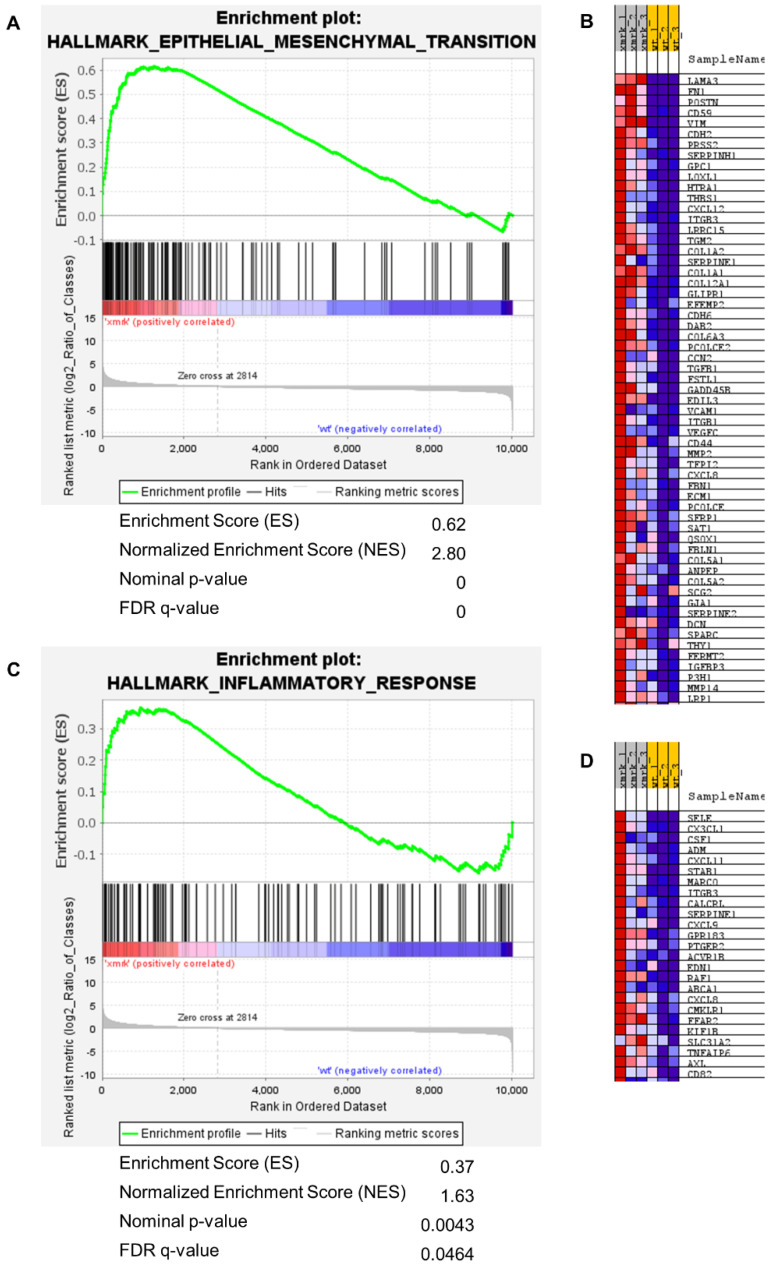
Gene set enrichment analysis of hallmark gene sets epithelial mesenchymal transition and inflammatory response. (**A**) GSEA summary plot of epithelial mesenchymal transition gene set comparing *xmrk* intestine to wt intestine. The gene set was highly enriched and upregulated: normalized enrichment score (NES) = 2.80, FDR *q*─value < 0.0001. (**B**) Heatmap of leading edge subset genes within the epithelial mesenchymal transition gene set (red: high expression; blue: low expression). (**C**) GSEA summary plot of inflammatory response gene set comparing *xmrk* intestine to wt intestine. The gene set was enriched and upregulated: NES = 1.63, FDR *q*─value = 0.0464. (**D**) Heatmap of leading edge subset genes within the inflammatory response gene set (red: high expression; blue: low expression).

Further gene set enrichment analysis was performed using the Molecular Signature Database (MSigDB) hallmark gene sets. The hallmark gene sets represent the well-defined biological processes by containing coherently expressed signatures from many MSigDB datasets [43]. Two hallmark gene sets, epithelial mesenchymal transition and inflammatory response, were highly enriched and upregulated. NES = 2.80, FDR *q*-value < 0.001 for epithelial mesenchymal transition (Figure 6A); NES = 1.63, FDR *q*-value < 0.05 for inflammatory response (Figure 6C). Leading edge genes that contributed most to the enrichment score were presented in heatmaps (Figure 6B,D). It is known that patients with inflammatory bowel disease usually develop intestinal fibrosis, which is marked by the activated epithelial-to-mesenchymal transition process [44]. Reactome GSEA showed the activation of pathways in the extracellular matrix organization, hemostasis and complement cascade (Table 1). Excessive deposition of extracellular matrix may lead to intestine fibrosis [45]. Fibrosis and increased hemostasis are known to be involved in inflammatory bowel disease [46,47]. The activation of complement underlies chronic inflammation. Overall, Reactome and hallmark gene sets enrichment analysis indicated that the intestine in liver tumor fish may resemble inflammatory bowel disease in human patients.

To test this hypothesis, human intestinal transcriptome of inflammatory bowel disease from GEO database, as well as the colon adenocarcinoma transcriptome from TCGA-TOAD project, were compared with the enriched genes (upregulated genes) of *xmrk* fish intestine (Table 2). Crohn’s disease and ulcerative colitis are the two major inflammatory bowel disease conditions. Crohn’s disease affects both the small and large intestines, whereas ulcerative colitis affects the colon and rectum. The positive NES value and FDR *q*-value < 0.05 indicated positive and significant enrichment of the *xmrk* intestine enriched genes in Crohn’s disease and ulcerative colitis of the colon. Crohn’s disease of the ileum also showed positive correlation with the *xmrk* intestine but not statistically significant. In comparison, colon adenocarcinoma was negatively correlated with the *xmrk* intestine. Thus, the GSEA comparison showed common gene signatures between the *xmrk* intestine and human inflammatory bowel disease.

### 3.6. Deregulation of Neutrophil-Related Genes and Intestinal-Function-Related Genes during HCC

**Figure 7:**
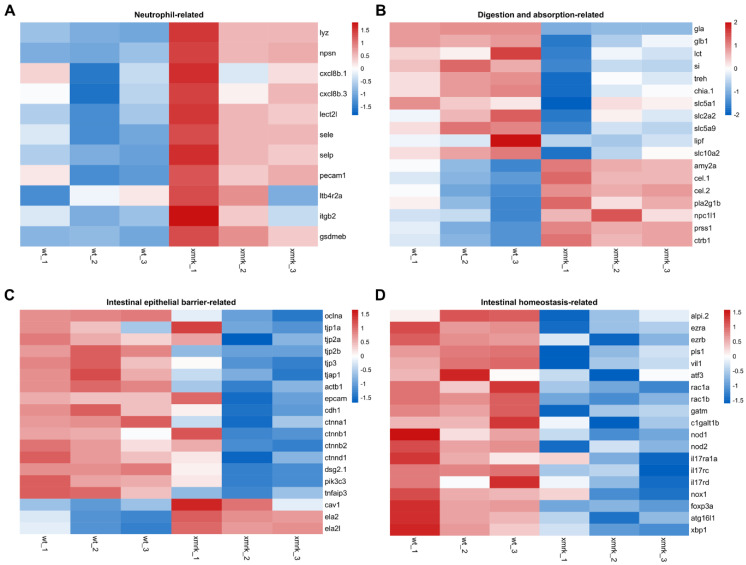
Differential expression profiles of genes related to neutrophils and intestine function. (**A**–**D**) Heatmap showing expression profile of neutrophil─related genes (**A**), digestion─ and absorption─related genes (**B**), intestinal epithelial barrier─related genes (**C**) and intestinal homeostasis─related genes (**D**). Heatmaps were generated with log─transformed TPM values, which were row scaled using z─scores, using the Heatmap module (v0.2.4) on the Hiplot platform (https://hiplot.com.cn, accessed on 4 April 2022).

To further study immune cell infiltration and intestine function in the *xmrk* intestine, the expression profiles of related genes were examined. Neutrophil-related genes included zebrafish neutrophil-specific marker genes, as well as genes important for neutrophil recruitment (Figure 7A). TPM values from RNA sequencing results were log transformed and z-score normalized across the six samples for each gene to generate the final heatmap. All genes presented in the heatmap were shown to have a higher expression in the *xmrk* samples compared to the wt, suggesting an overall accumulation of neutrophils in the *xmrk* intestine.

The expression profile of genes involved in the digestion and absorption process is shown in Figure 7B. These genes include digestive enzymes and solute carrier transporters for each nutrient group. The heatmap showed that these genes were all deregulated in the *xmrk* intestine. Genes involved in carbohydrate digestion and absorption were mostly downregulated, while peptide digestive enzymes were upregulated. On the other hand, there was a mix of up and downregulation for genes that participate in lipid digestion and transport. Overall, these data suggested a disruption in the processes of digestion and absorption of nutrients from food.

Intestinal epithelial cell barrier function was also examined, as it serves as an important barrier between commensal microbes, as well as infectious pathogens in the gut lumen and the internal enviroment of the intestine [48]. The expression profiles of genes involved in the formation and maintenance of cell junctions, which maintain epithelial cell layer integrity [49], are shown in Figure 7C. Non-cell junction genes that were shown to increase intestinal epithelial permeability when deregulated were also included. In the *xmrk* fish, there is a general downregulation in cell junction genes and an expected deregulation in non-cell junction genes that cause increased epithelial permeability, indicating a loss of epithelial cell barrier integrity in the *xmrk* intestine.

Lastly, genes involved in general intestinal homeostasis were examined. Genes that played roles in epithelial and microvilli organization, regulation and maintenance of gut microbiome, epithelial repair, as well as autophagy and ER stress response were examined (Figure 7D). As shown in the heatmap (Figure 7D), all genes were downregulated in the *xmrk* intestine, suggesting a breakdown in intestinal homeostasis under the HCC condition.

## 4. Discussion

In this study, we systematically evaluated the effect of liver tumor on the intestine and demonstrated the progressive disruption to the intestine during liver tumor progression using the zebrafish model. We showed the structural disorganization of intestine through histological examination. Intestinal architecture disruption is present in many intestinal disorders and conditions. Changes to bowel wall thickness occur under conditions of tumorigenesis, ischemia, infection and inflammation [50,51]. Intestinal epithelial ulceration and erosion have been reported in human IBD biopsies, as well as mice models [52,53]. Similar architectural changes have been observed in zebrafish intestinal disease models, especially in models of IBD. One of the first zebrafish models of intestinal inflammation was established by inducing enterocolitis via intrarectal hapten oxazolone injection in adult zebrafish. Inflammation developed in the intestine of oxazolone-induced fish, and histological examination showed bowel wall thickening and villus atrophy in the inflamed intestines [36]. Trinitrobenzene-sulfonic acid (TNBS)-induced colitis in adult zebrafish showed ulcerations and sloughing of villi, suggesting cell damage and disruption of epithelial integrity [54,55]. The intestine is a highly complex organ, and proper architecture is required for optimal functioning. For instance, villi ulceration and erosion involves epithelial cell death [56] and would not only reduce the intestine’s capacity for absorption of nutrients, but exposure of the lamina propria to the intestinal lumen would also greatly increase the risk of invasion by commensal and pathogenic microbes. Based on the gene expression profiles shown in Figure 7B–D, normal intestine function was affected by HCC. It is likely that the severe architectural disruption seen in the *xmrk* intestine plays a considerable role in altering intestine function.

Goblet cells are a population of epithelial cells in the intestine that secrete mucins, which are glycoproteins that form a mucus blanket covering the epithelial cell layer. The mucus layer functions as an additional protective barrier that helps prevent pathogens and foreign antigens from invading the intestinal tissue. These specialized cells and the mucus they secrete also participate in modulating the intestinal immune system. Moreover, it is known that immune cells, such as macrophages and T cells, as well as various cytokines present in the lamina propria, regulate goblet cell function and differentiation [40,57]. Disruption in goblet cell proliferation, differentiation and function leads to intestinal mucosal barrier dysfunction, which has been shown to occur in both intestinal disorders and extra-intestinal disorders, such as diabetes and non-alcoholic fatty liver disease [58]. Goblet cell hyperplasia and the accompanying increase in mucin production usually occur in parasitic and bacterial infections, as well as in animal models of intestinal inflammation [41,59]. This phenotype is also seen in various zebrafish IBD models, such as the dextran sodium sulfate (DSS)- and TNBS-induced fish [60,61]. Though not much research has been carried out on goblet cell size and its biological significance, large goblet cells have been considered mature, and smaller cells have been associated with goblet cell immaturity in both zebrafish and mice [62,63]. As we observed an increase in goblet cell number and a reduction in goblet cell size in the *xmrk* intestine, it is possible that goblet cell proliferation and/or apoptosis is dysregulated, and the cells fail to fully mature under the HCC condition. It has been shown that bile acid affects goblet cells and mucus secretion by altering gut microbiota [64], and intestinal metabolites, including those produced by microbiota, in turn regulate bile acid synthesis [8]. Furthermore, altered bile acid levels and modified bile acids are present in liver diseases [65,66], and evidence strongly suggests that elevated levels of bile acids can contribute to liver carcinogenesis, either directly or indirectly, via the gut microbiome [67,68]. The multiway crosstalk between these mechanisms can come together to potentially cause a loop of dysregulation across the gut–liver axis, exacerbating the effect of HCC on the liver, intestine and other gastrointestinal tract and related organs.

The gut immune system is an intricate and sophisticated environment that contributes to the maintenance of intestinal homeostasis. Epithelial cells and gut resident-immune cells produce and respond to chemical signals, such as cytokines and chemokines, resulting in the secretion of substances, such as antimicrobial peptides, antibodies or mucins, that help kill and expel invading microorganisms from the body. Commensal microbiota in the gut also works in tandem with the host immune system to regulate homeostasis, by playing important roles in processes, such as nutrient metabolism and proper gastrointestinal development [69]. Though protective and beneficial in mucosal homeostasis and wound healing, excessive gut infiltration of immune cells, such as eosinophils and neutrophils, is associated with inflammatory conditions in the intestine [70,71]. Eosinophils and neutrophils are leukocytes that accumulate in the gut and are activated in times of infection and inflammation. Upon stimulation, both cell types release antimicrobial proteins, extracellular traps and signaling molecules that protect the host mucosa [72,73]. However, in IBD conditions, eosinophil and neutrophil infiltration and activation is found to be dysregulated and excessive, resulting in exacerbating gut epithelial cell damage and increase in mucosal barrier permeability [70,72,74]. There might even be crosstalk between the two cell types, as Il-8 induces neutrophil chemotaxis and activation [75], even in the zebrafish [76], and eosinophils have been shown to be capable of producing the cytokine and chemotactically respond to Il-8 [72,77,78].

Transcriptomic analysis showed substantial changes in the intestine under the liver tumor condition. Intestinal function was apparently disrupted in the *xmrk* fish, based on the deregulation of genes involved in digestion and absorption of nutrients from food, downregulation of genes maintaining epithelial cell barrier integrity, as well as downregulation of genes involved in general intestinal homeostasis. The activation of pathways in inflammation, ECM organization, EMT and hemostasis, which were found in the intestine of the *xmrk* fish, have also been reported in the inflammatory bowel disease in human patients [44,46,79]. Our GSEA analysis suggested common gene signatures between the *xmrk* intestine and human inflammatory bowel disease. The association of inflammatory bowel disease with liver disorders, such as hepatobiliary cancer, has been noticed in previous clinical studies [80,81,82]. Studies using the mouse model have shown that tumors can induce inflammation and complex DNA damage in distant tissues, such as the intestine [5,6].

Numerous studies have highlighted the association between gut microbial dysbiosis and liver cancer [10]. The chronic inflammation and change of bile acid secretion of liver tumor could cause changes in gut microbiota through the gut–liver axis [83]. We attempted to examine the gut microbiota in the zebrafish liver tumor model through 16s rRNA sequencing. However, the addition of the antibiotic doxycycline in fish water affected the microbial species in the gut, resulting in low species richness and poor species evenness in both the wt and *xmrk* fish. No significant difference of microbiota was observed, most likely masked by the effect of antibiotic. Therefore, it is worthwhile to study the gut microbiota in another liver tumor model that does not use the doxycycline inducible system. Studies have shown that gut microbiota play important roles in liver cancer development. The gut dysbiosis and the resulting gut leakiness in chronic liver disease and liver cancer may in turn promote tumorigenesis through bacterial metabolites and inflammation [84]. Modulating gut microbiota has been proposed as a treatment strategy for HCC, which could be tested in the zebrafish liver tumor model for screening of such prebiotics and probiotics.

## 5. Conclusions

Our present study shows the significant and progressive disruption to intestinal organization during liver tumorigenesis using the zebrafish liver tumor model. The intestine under the liver tumor condition resembles the inflammatory intestine from animal models and human samples, as suggested by the histological and transcriptomic analysis. This study provides the first demonstration of crosstalk between the liver tumor and the intestine in animal models. We propose that intestinal inflammation may be a potential target for treating cancer cachexia in HCC patients.

## Figures and Tables

**Table 1:** Significantly enriched and deregulated Reactome pathways from GSEA analysis.

Category	Pathway Name	NES	FDR *q*-Value
Extracellular Matrix Organization	EXTRACELLULAR_MATRIX_ORGANIZATION	2.78	0
	ECM_PROTEOGLYCANS	2.62	0
	ELASTIC_FIBRE_FORMATION	2.54	1.94 × 10^–4^
	MOLECULES_ASSOCIATED_WITH_ELASTIC_FIBRES	2.39	4.60 × 10^–4^
	COLLAGEN_FORMATION	1.92	3.33 × 10^–2^
	ASSEMBLY_OF_COLLAGEN_FIBRILS_AND_OTHER_MULTIMERIC_STRUCTURES	2.02	2.12 × 10^–2^
	NON_INTEGRIN_MEMBRANE_ECM_INTERACTIONS	2.46	2.33 × 10^–4^
	SYNDECAN_INTERACTIONS	2.01	2.21 × 10^–2^
	ACTIVATION_OF_MATRIX_METALLOPROTEINASES	2.43	2.04 × 10^–4^
	DEGRADATION_OF_THE_EXTRACELLULAR_MATRIX	2.37	5.83 × 10^–4^
	LAMININ_INTERACTIONS	2.29	1.59 × 10^–3^
	INTEGRIN_CELL_SURFACE_INTERACTIONS	2.28	1.78 × 10^–3^
Hemostasis	PLATELET_AGGREGATION_PLUG_FORMATION	2.53	1.55 × 10^–4^
	RESPONSE_TO_ELEVATED_PLATELET_CYTOSOLIC_CA2	1.98	2.53 × 10^–2^
	CELL_SURFACE_INTERACTIONS_AT_THE_VASCULAR_WALL	1.96	2.91 × 10^–2^
	FORMATION_OF_FIBRIN_CLOT_CLOTTING_CASCADE	1.92	3.41 × 10^–2^
Cell Cycle	CELL_CYCLE_CHECKPOINTS	–1.98	1.11 × 10^–3^
	STABILIZATION_OF_P53	–2.42	0
	G1_S_DNA_DAMAGE_CHECKPOINTS	–2.38	0
	G2_M_CHECKPOINTS	–2.30	4.83 × 10^–5^
	CELL_CYCLE_MITOTIC	–1.90	3.43 × 10^–3^
	MITOTIC_G1_PHASE_AND_G1_S_TRANSITION	–2.31	5.31 × 10^–5^
	S_PHASE	–2.28	4.25 × 10^–5^
	MITOTIC_G2_G2_M_PHASES	–1.83	6.75 × 10^–3^
	M_PHASE	–1.73	1.83 × 10^–2^
	MITOTIC_METAPHASE_AND_ANAPHASE	–1.76	1.42 × 10^–2^
	CHROMOSOME_MAINTENANCE	–1.96	1.32 × 10^–3^
	TELOMERE_MAINTENANCE	–1.91	2.70 × 10^–3^
DNA Replication	DNA_REPLICATION	–2.33	5.90 × 10^–5^
	DNA_REPLICATION_PRE_INITIATION	–2.39	0
	SYNTHESIS_OF_DNA	–2.37	0
	DNA_STRAND_ELONGATION	–2.20	3.03 × 10^–5^
	SWITCHING_OF_ORIGINS_TO_A_POST_REPLICATIVE_STATE	–2.11	1.94 × 10^–4^
DNA Repair	DNA_REPAIR	–1.69	2.78 × 10^–2^
	TRANSCRIPTION_COUPLED_NUCLEOTIDE_EXCISION_REPAIR_TC_NER	–1.67	3.13 × 10^–2^
	BASE_EXCISION_REPAIR	–1.80	9.68 × 10^–3^
	DNA_DOUBLE_STRAND_BREAK_REPAIR	–1.77	1.30 × 10^–2^
	HOMOLOGY_DIRECTED_REPAIR	–1.74	1.63 × 10^–2^
	DNA_DAMAGE_BYPASS	–1.80	9.04 × 10^–3^
Transcription	RNA_POLYMERASE_II_TRANSCRIPTION_TERMINATION	–1.74	1.64 × 10^–2^
	REGULATION_OF_RUNX2_EXPRESSION_AND_ACTIVITY	–2.18	2.79 × 10^–5^
	REGULATION_OF_RUNX3_EXPRESSION_AND_ACTIVITY	–2.30	5.06 × 10^–5^
	RUNX1_REGULATES_TRANSCRIPTION_OF_GENES_INVOLVED_IN_DIFFERENTIATION_OF_HSCS	–2.04	5.20 × 10^–4^
	TP53_REGULATES_TRANSCRIPTION_OF_CELL_DEATH_GENES	–1.76	1.48 × 10^–2^
	GENE_SILENCING_BY_RNA	–1.66	3.18 × 10^–2^
Metabolism of RNA	REGULATION_OF_MRNA_STABILITY_BY_PROTEINS_THAT_BIND_AU_RICH_ELEMENTS	–2.10	2.31 × 10^–4^
	PROCESSING_OF_CAPPED_INTRON_CONTAINING_PRE_MRNA	–1.97	1.27 × 10^–3^
	MRNA_SPLICING	–1.93	2.04 × 10^–3^
	TRANSPORT_OF_MATURE_TRANSCRIPT_TO_CYTOPLASM	–1.83	6.69 × 10^–3^
	SNRNP_ASSEMBLY	–1.67	3.01 × 10^–2^
Metabolism of Proteins	TRANSLATION	–1.84	6.77 × 10^–3^
	MITOCHONDRIAL_TRANSLATION	–2.41	0
	DEUBIQUITINATION	–1.83	6.67 × 10^–3^
	NEDDYLATION	–1.75	1.64 × 10^–2^
	ASPARAGINE_N_LINKED_GLYCOSYLATION	–1.61	4.66 × 10^–2^
Immune System	COMPLEMENT_CASCADE	1.96	2.87 × 10^–2^
	INITIAL_TRIGGERING_OF_COMPLEMENT	2.02	2.15 × 10^–2^
	TNFR2_NON_CANONICAL_NF_KB_PATHWAY	–2.29	4.42 × 10^–5^
	INTERLEUKIN_1_SIGNALING	–2.22	3.32 × 10^–5^
	INTERLEUKIN_12_FAMILY_SIGNALING	–1.72	2.10 × 10^–2^
	C_TYPE_LECTIN_RECEPTORS_CLRS	–2.08	2.55 × 10^–4^
	FC_EPSILON_RECEPTOR_FCERI_SIGNALING	–2.12	1.98 × 10^–4^
	DDX58_IFIH1_MEDIATED_INDUCTION_OF_INTERFERON_ALPHA_BETA	–1.67	3.15 × 10^–2^
	ROS_AND_RNS_PRODUCTION_IN_PHAGOCYTES	–1.66	3.30 × 10^–2^
	SIGNALING_BY_THE_B_CELL_RECEPTOR_BCR	–2.23	3.43 × 10^–5^
	CLASS_I_MHC_MEDIATED_ANTIGEN_PROCESSING_PRESENTATION	–1.66	3.31 × 10^–2^
Metabolism	CHOLESTEROL_BIOSYNTHESIS	2.56	2.59 × 10^–4^
	BIOLOGICAL_OXIDATIONS	1.90	3.71 × 10^–2^
	METABOLISM_OF_POLYAMINES	–2.28	4.08 × 10^–5^
	METABOLISM_OF_COFACTORS	–1.84	6.70 × 10^–3^
	THE_CITRIC_ACID_TCA_CYCLE_AND_RESPIRATORY_ELECTRON_TRANSPORT	–1.72	2.12 × 10^–2^
	RESPIRATORY_ELECTRON_TRANSPORT	–1.95	1.75 × 10^–3^
	COMPLEX_I_BIOGENESIS	–1.78	1.15 × 10^–2^
	CITRIC_ACID_CYCLE_TCA_CYCLE	–1.62	4.30 × 10^–2^
Protein Localization, Transport of Small Molecules	PROTEIN_LOCALIZATION	–1.81	8.57 × 10^–3^
	MITOCHONDRIAL_PROTEIN_IMPORT	–2.08	2.64 × 10^–4^
	ABC_FAMILY_PROTEINS_MEDIATED_TRANSPORT	–2.12	2.02 × 10^–4^
	PLASMA_LIPOPROTEIN_CLEARANCE	–1.63	4.09 × 10^–2^
Cellular Responses to Stimuli	CELLULAR_RESPONSE_TO_HYPOXIA	–2.48	0
	HSP90_CHAPERONE_CYCLE_FOR_STEROID_HORMONE_RECEPTORS_SHR_IN_THE_PRESENCE_OF_LIGAND	–1.86	4.85 × 10^–3^
	CELLULAR_RESPONSE_TO_CHEMICAL_STRESS	–1.83	7.10 × 10^–3^
	HSF1_ACTIVATION	–1.68	2.93 × 10^–2^
	ATTENUATION_PHASE	–1.60	4.95 × 10^–2^
Signal Transduction	GPCR_LIGAND_BINDING	2.35	8.28 × 10^–4^
	CLASS_A_1_RHODOPSIN_LIKE_RECEPTORS	2.48	2.72 × 10^–4^
	PEPTIDE_LIGAND_BINDING_RECEPTORS	2.28	1.80 × 10^–3^
	MET_PROMOTES_CELL_MOTILITY	2.26	2.11 × 10^–3^
	INTEGRIN_SIGNALING	2.25	2.18 × 10^–3^
	DEGRADATION_OF_AXIN	–2.40	0
	BETA_CATENIN_INDEPENDENT_WNT_SIGNALING	–2.03	5.47 × 10^–4^
	DEGRADATION_OF_BETA_CATENIN_BY_THE_DESTRUCTION_COMPLEX	–2.16	1.08 × 10^–4^
	DEGRADATION_OF_DVL	–2.39	0
	SIGNALING_BY_HEDGEHOG	–1.91	2.82 × 10^–3^
	HEDGEHOG_LIGAND_BIOGENESIS	–2.39	0
	SIGNALING_BY_NOTCH4	–2.07	3.58 × 10^–4^
	MAPK6_MAPK4_SIGNALING	–2.14	1.29 × 10^–4^
	REGULATION_OF_RAS_BY_GAPS	–2.27	3.79 × 10^–5^
	REGULATION_OF_PTEN_STABILITY_AND_ACTIVITY	–2.22	3.22 × 10^–5^

**Table 2:** GSEA identification of similar gene signatures between *xmrk* fish intestine and human intestinal disease datasets.

GEO/TCGA Acession	Human Intestine Dataset	Enriched Genes in *xmrk* Intestine	
		NES	FDR *p*-Value
GSE165512	Ulcerative colitis, colon	2.2856	<0.001
GSE165512	Crohn’s disease, colon	2.2315	<0.001
GSE165512	Crohn’s disease, ileum	0.6574	0.644
TCGA-COAD	Colon adenocarcinoma	–1.4089	<0.001

## Data Availability

The data presented in this study are available on request from the corresponding author.

## Supplementary Materials

The following are available online at www.mdpi.com/article/10.3390/cells11111810/s1, Figure S1: Severity of intestine phenotype after 4 weeks of HCC induction, Table S1: Statistics for reads filtering and mapping, Table S2: RNA-seq gene expression in TPM, Table S3: List of differentially expressed genes.
