# Supplementary material for: Systematic Characterization of the Disruption of Intestine during Liver Tumor Progression in the xmrk Oncogene Transgenic Zebrafish Model
Source: Cells. 2022 May 31;11(11):1810. doi: 10.3390/cells11111810 (PMC9180660; doi:10.3390/cells11111810)
Supplement: Supplementary file 1 [file cells-11-01810-s001.zip › cells-1743691-supplementary/xmrk intestine manuscript _ supplementary data _ submitted to Cells.pdf]

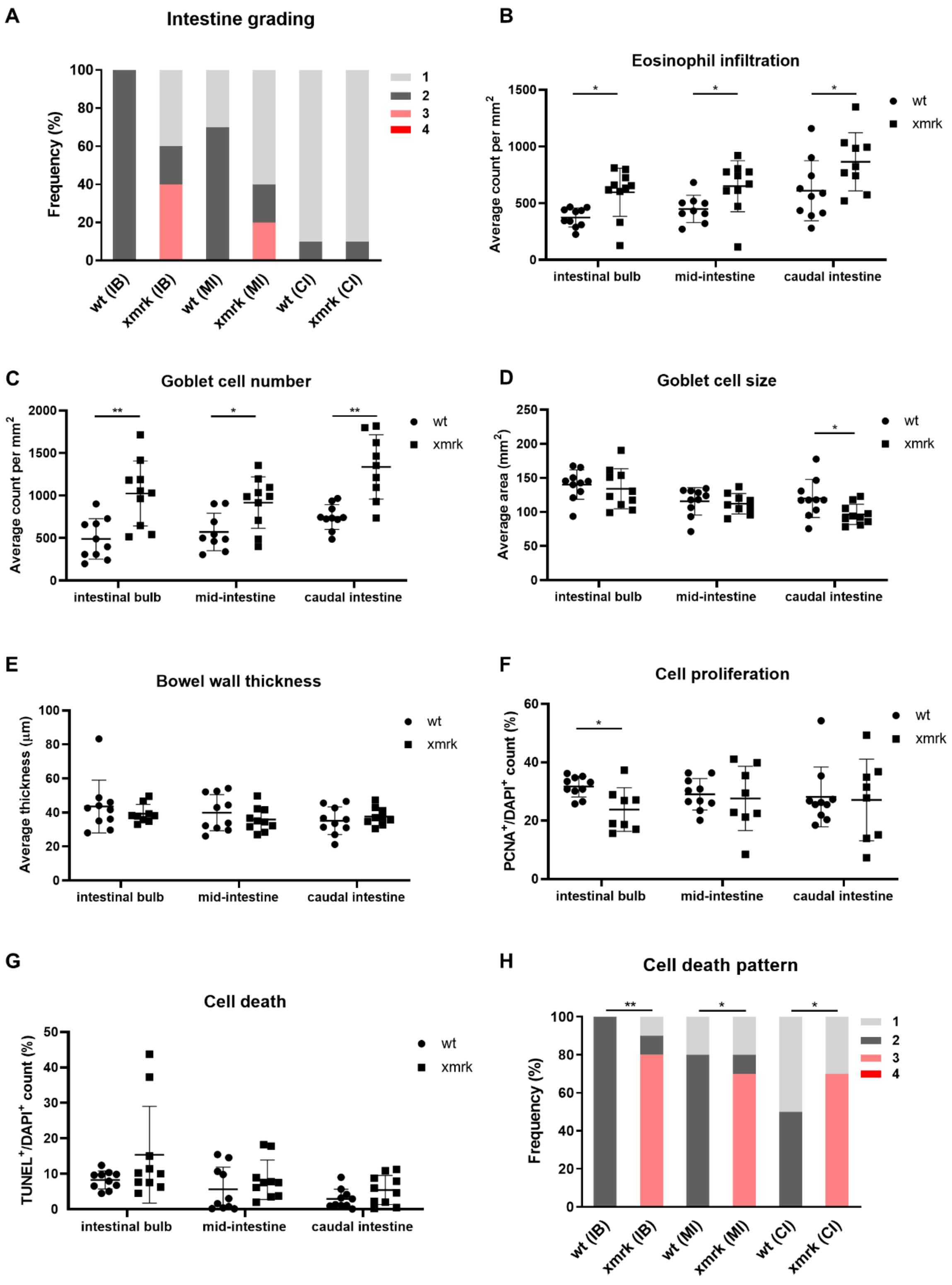


**Figure S1.** Severity of intestine phenotype after 4 weeks of HCC induction. (A-H) Quantification of intestinal grading severity (A), eosinophil counts (B), goblet cell counts (C), goblet cell size (D), bowel wall thickness (E), cell proliferation (F), cell death (G), and cell death pattern (H) in wt vs *xmrk* intestine. IB: intestinal bulb; MI: mid-intestine; CI: caudal intestine. **P* <0.05, ***P* <0.01

**Table S1. Statistics for reads filtering and mapping**

| Sample Name | wt_1 | wt_2 | wt_3 | xmrk_1 | xmrk_2 | xmrk_3 |
|---|---|---|---|---|---|---|
| Total Raw Reads (M) | 106.92 | 101.66 | 103.42 | 103.42 | 101.66 | 99.91 |
| Total Clean Reads (M) | 89.05 | 88.98 | 89.25 | 89.38 | 88.49 | 87.99 |
| Clean Reads Ratio (%) | 83.29 | 87.53 | 86.3 | 86.43 | 87.04 | 88.07 |
| Total Mapping(%) | 62.78 | 64.09 | 66.07 | 59.48 | 68 | 70.87 |
| Uniquely Mapping(%) | 58.62 | 60.08 | 62.38 | 54.24 | 62.74 | 65.3 |

**Table S2. RNA-seq gene expression in TPM** (in separate excel file)

**Table S3. List of differentially expressed genes** (in separate excel file)
